# Prehospital control of life-threatening truncal and junctional haemorrhage is the ultimate challenge in optimizing trauma care; a review of treatment options and their applicability in the civilian trauma setting

**DOI:** 10.1186/s13049-016-0301-9

**Published:** 2016-09-13

**Authors:** S. E. van Oostendorp, E. C. T. H. Tan, L. M. G. Geeraedts

**Affiliations:** 1 Department of Trauma Surgery, VU University Medical Center, P.O. Box 7057, 1007 MB Amsterdam, The Netherlands; 2 Department of Trauma Surgery and Helicopter Emergency Medical Service, Radboud University Medical Center, Nijmegen, The Netherlands; 3 Royal Netherlands Army, Utrecht, The Netherlands

**Keywords:** Trauma, Haemorrhage, Exsanguination, Intervention, Prehospital, Junctional, Truncal, Bleeding

## Abstract

**Introduction:**

Exsanguination following trauma is potentially preventable. Extremity tourniquets have been successfully implemented in military and civilian prehospital care. Prehospital control of bleeding from the torso and junctional area’s remains challenging but offers a great potential to improve survival rates. This review aims to provide an overview of potential treatment options in both clinical as preclinical state of research on truncal and junctional bleeding. Since many options have been developed for application in the military primarily, translation to the civilian situation is discussed.

**Methods:**

Medline (via Pubmed) and Embase were searched to identify known and potential prehospital treatment options. Search terms were|: *haemorrhage/hemorrhage, exsanguination, junctional, truncal, intra-abdominal, intrathoracic, intervention, haemostasis/hemostasis, prehospital, en route, junctional tourniquet, REBOA, resuscitative thoracotomy, emergency thoracotomy, pelvic binder, pelvic sheet, circumferential.* Treatment options were listed per anatomical site: axilla, groin, thorax, abdomen and pelvis Also, the available evidence was graded in (pre) clinical stadia of research.

**Results:**

Identified treatment options were wound clamps, injectable haemostatic sponges, pelvic circumferential stabilizers, resuscitative thoracotomy, resuscitative endovascular balloon occlusion of the aorta (REBOA), intra-abdominal gas insufflation, intra-abdominal self-expanding foam, junctional and truncal tourniquets. A total of 70 papers on these aforementioned options was retrieved. No clinical reports on injectable haemostatic sponges, intra-abdominal insufflation or self-expanding foam injections and one type of junctional tourniquets were available.

**Conclusion:**

Options to stop truncal and junctional traumatic haemorrhage in the prehospital arena are evolving and may offer a potentially great survival advantage. Because of differences in injury pattern, time to definitive care, different prehospital scenario’s and level of proficiency of care providers; successful translation of various military applications to the civilian situation has to be awaited. Overall, the level of evidence on the retrieved adjuncts is extremely low.

## Background

Haemorrhage due to trauma is the leading preventable cause of death in the military setting, accounting up tot to 90 % of potentially preventable deaths [1, 2]. In the civilian setting, it is the second most leading cause of death in trauma patients, studies report: 26–40 % [3, 4]. Only head injury is considered more lethal [3, 4]. Fifty-six per cent up to 87 % for respectively civilian and combat-related mortality caused by traumatic haemorrhage occurs before reaching definitive care [2, 5, 6]. So, early (e.g., prehospital) haemorrhage control allowing for bridging to definitive surgical care, may yield a large survival advantage.

Life-threatening haemorrhage is a time dependent ‘disease’ in which the duration of ongoing bleeding may lead to either death or, in case of initial survival and subsequent massive transfusion, to possible sepsis and/or multi-organ failure [7–9]. Haemorrhage control, among shock resuscitation and prevention of trauma-induced coagulopathy, are the mainstays of treatment of imminent exsanguination in the prehospital arena as well as in the definitive care facility [9].

Treatment options for ongoing haemorrhage in the prehospital arena are limited. In case of blunt trauma and closed extremity injuries, (traction) splinting of limbs and/or stabilizing the pelvis reduces blood loss until definitive care [10]. In open fractures caused by blunt trauma and in extremity injuries resulting from penetrating trauma or blast; external blood loss is limited by the use of extremity tourniquets, with excellent results in the military [11, 12] and civilian [13–16] setting. Extreme nasopharyngeal and/or oropharyngeal bleeding can be countered by gauze packing and/or improvised *Bellocq*-tamponade with Foley-catheters [17, 18]. Hemostatic suturing, direct digital pressure and the use of pressure bandages will prevent ongoing blood loss from external wounds such as scalp lacerations [19–21]. Moreover, for the last 15 years [22] there has been an ongoing development of haemostatic gauze dressings that can be used as an adjunct to compression in stopping major blood loss from external wounds. Their applicability and efficacy have been published in recent review articles [22–24]. As with the tourniquet, the use of haemostatic gauze dressings has found its way from the battlefield to application in the civilian prehospital setting [25, 26], although wide-spread implication of both treatment options has not been achieved [27, 28]. However, through the *Hartford Consensus* and in the context of prehospital care in mass shootings and/or bomb explosions in the civilian situation, application of tourniquets and haemostatic gauze dressings by non-(para) medical responders and lay persons is promoted to stop bleeding early [29, 30]. Half day training courses for these immediate responders have been set up [31].

In the prehospital arena, stopping major bleeding from anatomical sites where the application of an extremity tourniquet or pressure bandage is not feasible, remains the greatest challenge. Bleeding from the so-called junctional area’s: axillae, groins and neck are compressible. Direct digital pressure combined with haemostatic gauze dressings are temporizing measures but may not be feasible under hectic, tactical prehospital circumstances and/or prolonged extraction and transport procedures.

For non-compressible bleeding from the trunc: options for (temporary) control of intrathoracic, intra-abdominal, and intrapelvic haemorrhage are extremely limited in the prehospital arena and haemorrhage control in these cases may demand expert surgical approach [32].

The aim of this review is to provide an overview of modern (experimental) treatment options for control of junctional and truncal haemorrhage in the prehospital arena. Also, since most developments are ensuing from necessities in the battlefield situations, the applicability of the treatment options are discussed in the light of the civilian setting and especially in the context of differences in patient demographics, trauma mechanism and prehospital situations.

## Methods

Exploration of existing reports in the literature was done by a search of Medline (via Pubmed) and Embase at December 22th 2015 without restriction to publication date. Papers eligible for inclusion had to be written in English. No restriction was applied to the stage of research, i.e., articles on both clinical and preclinical research were eligible. The search was performed of the following terms in various combinations: *haemorrhage/hemorrhage, exsanguination, junctional, truncal, intra-abdominal, intrathoracic, intervention, haemostasis/hemostasis, prehospital, en route, junctional tourniquet, REBOA, resuscitative thoracotomy, emergency thoracotomy, pelvic binder, pelvic sheet, circumferential*. Initially, all study reports of interventions regardless of design (i.e., trials, case-series, case-reports and reviews) were included. All abstracts were assessed for the potential for prehospital applicability of the intervention in case of haemorrhage by experienced prehospital trauma care providers (ET and LG). References of the reports were scanned to retrieve additional studies.

The focus of this review was on reports of prehospital or experimental (i.e., cadaveric or animal) prehospital procedures. However, if such reports on certain body areas were scarce, we included recent studies reporting in-hospital emergency department procedures that potentially could be applied prehospitally. The interventions of the selected studies were allocated to 5 anatomical areas of interest: junctional: 1) axilla, 2) groin and truncal 3) chest, 4) abdomen and 5) pelvis The results are described per anatomical area and presented in a table ordered by (pre) clinical stadium of research. Finally, an appraisal of potential applicability in the prehospital arena is provided by listing pro’s and con’s as discussed in meetings by the authors of this review.

## Results

### Junctional haemorrhage

Junctional haemorrhage is defined as bleeding from a junction of the torso to the extremities, i.e., the base of the neck, shoulder, axilla, perineum, buttocks, gluteal area and the groin [33]. A study of casualties in the U.S. combat forces from 2001 to 2011, noted that 17.5 % of potentially preventable prehospital deaths resulted from junctional haemorrhage [2]. No reports on the incidence of junctional haemorrhage in the civilian situation were found.

#### Junctional haemorrhage – axilla- direct pressure

The axilla is a vulnerable spot, poorly protected by armoured vests in the military setting. An extremity tourniquet is considered as treatment of first resort in haemorrhage of the upper extremity. If bleeding persists despite an adequately applied tourniquet or the site of bleeding is too proximal to apply a tourniquet, military guidelines advocate to start with a haemostatic gauze in combination with direct pressure [33]. The prehospital use and efficacy of haemostatic gauzes has been reviewed extensively [22–24] and will not be discussed. After animal-model testing [34], the FDA approved XSTAT-30™ (RevMedx, Wilsonville, Ore), a syringe-like device containing dozens of small compressed chitosan covered cellulose sponges that can be injected in junctional bleedings and secured with regular bandages. Intra-thoracic, −pelvic or –abdominal use are contra-indicated [35]. No clinical use was yet reported at the time of this review.

#### Junctional haemorrhage – axilla- junctional tourniquets

A downside of applying direct pressure is that a care provider is no longer available to perform other interventions. In the military, due to tactical circumstances and longer retrieval times, this has led to a request for mechanical devices to free the hands of the medical care provider.

Four junctional tourniquets which can be used in case of junctional haemorrhage are the Combat Ready Clamp (CRoC)™, Junctional Emergency Tool (JETT)™, SAM Junctional Tourniquet (SAM-JT)™ and Abdominal Aortic Junctional Tourniquet (AAJT)™ [33]. Simultaneous use of haemostatic dressings and (junctional) tourniquets may work synergistic in controlling haemorrhage [33, 36].

**Fig. 1:**
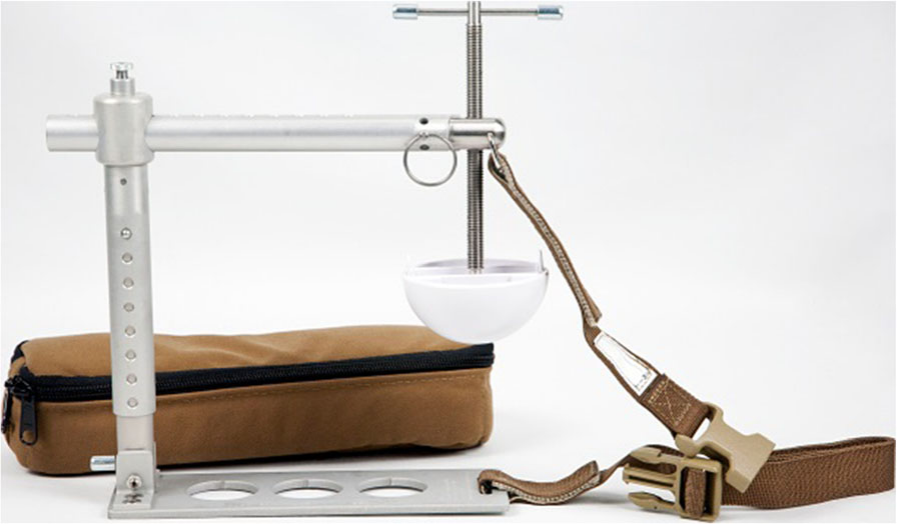
CRoC™. Https://combatmedicalsystems.wordpress.com/2013/05/06/combat-ready-clamp-croctm-makes-tactical-medicine-history/

The CRoC™ has been FDA-cleared for axillary haemorrhage and has shown to be effective in a cadaver model [37]. It is a device which consists of a stamp compressing the targeted area underneath. See Fig. 1. It has been reflected that the CRoC™ is heavy and assembling takes too long (1–2 min) [38]. However, in civilian prehospital care, a ready-to-use pre-assembled unit can be stored in an ambulance or helicopter [33].

At the time of this review no studies on the use of JETT™ or SAM-JT™ junctional tourniquet for axillary bleeding were available.

The AAJT™ has been successfully used in the civilian setting in case of a gunshot wound to the axilla [39]. The wedge-formed bladder was positioned in the axilla and the device strapped around the torso. No further bleeding from the axilla was seen after inflating the bladder up to 250 mmHg pressure. Radiographic imaging showed some compression of the left thorax, but ventilator inspiratory pressures were normal [39]. An observational trial in human volunteers showed successful eliminated blood flow in the brachial artery in axillary application of the AAJT™ using a mean pressure of 168 mmHg. Doppler measurement showed complete elimination of flow and spontaneous recovery of flow when the AAJT™ was deflated [40].

#### Junctional haemorrhage – axilla- wound clamp

**Fig. 2:**
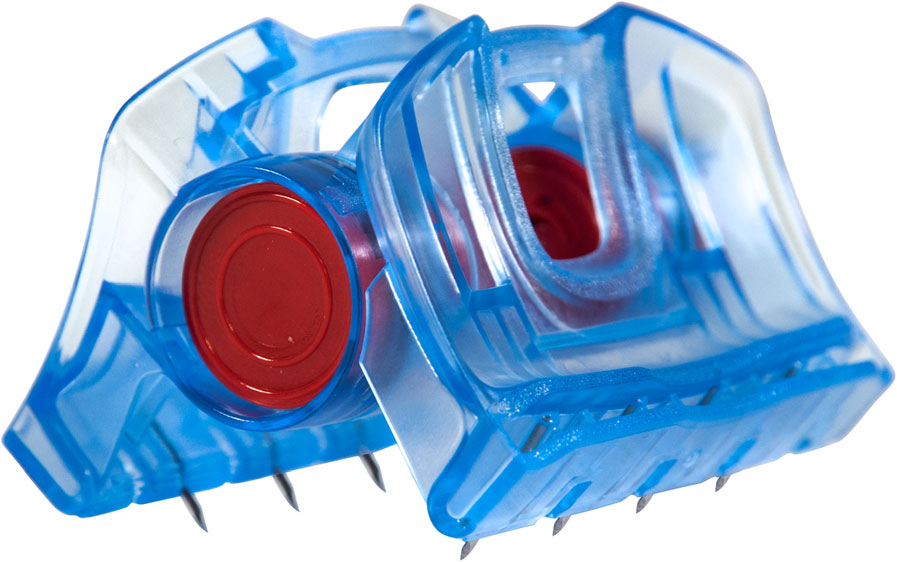
iTClamp™. Image provided by manufacturer

When a junctional tourniquet is not available, or will take too much time to apply and/or when maintaining direct pressure is not an option, the use of the iTClamp™ (Innovative Trauma Care Inc., Edmonton, Alberta, Canada) may be considered. The iTClamp™ is a mechanical clamp which looks like a hair clamp with several small needles that seals a wound by approximating the wound edges of the overlying skin firmly, creating a compartment in which the bleeding potentially tamponades. See Fig. 2. Several reports of field use exist [41, 42]. See Table 1. With the iTClamp™ haemorrhage control was gained in 9 out of 10 applications for venous or arterial and venous combined origin in scalp (*n* = 7), neck, chest wall, and lacerations from open femoral fractures. The clamp failed to control haemorrhage from a combined carotid and vertebral arterial laceration in the neck. By packing the wound with haemostatic gauze, reapplication of the iTClamp™ and direct pressure haemorrhage control was achieved [42].

**Table 1:** Overview of retrieved studies covering the identified prehospital treatment options, ordered by anatomical site, device and stage of (pre-clinical) research

Site	Device	Clinical			Preclinical			Physician required?
		Prehospital civilian	Prehospital military	Inhospital	Volunteer	Cadaver/manikin	Animal	
Axilla	CRoC	-	-	-	-	[37]	-	No
	JETT	-	-	-	-	-	-	No
	SAM-JT	-	-	-	-	-	-	No
	AAJT	[39]	-	-	[40]	-	-	No
	iTClamp	-	-	-	-	-	-	No
	Xstat-30	-	-	-	-	-	[34]	No
Groin	CRoC	-	[45]	-	[43]	[47, 128, 129]	[38, 130]	No
	JETT	-	-	-	[43]	[47, 129]	-	No
	SAM-JT	-	[48]	-	[43]	[129]	-	No
	AAJT-Groin	-	[50]	-	[40]	-	-	No
	AAJT-Truncal	-	[49]	-	[43, 44, 46]	[129]		No
	iTClamp	[53]	[41]	-	-	[52]	[51, 131]	No
	Xstat-30	-	-	-	-	-	[34]	No
	REBOA	-	-	[54, 98]	-	-	[64, 132]	Yes
Abdominal	REBOA	-	-	[54, 61, 62, 97, 98]	-	-	[57, 58, 63, 96, 113]	Yes
	Resuscitative thoracotomy	[66, 70, 71]	-	[62, 76]	-	-	-	Yes
	Insufflation	-	-	-	-	-	[84–88]	No
	ResQFoam	-	-	-	-	[94]	[82, 83, 89–92]	No
Thorax	Resuscitative thoracotomy	[66, 69–72, 74]	-	[76]	-	-	-	Yes
	REBOA	-	-	-	-	-	-	Yes
Pelvis	Sheet	-	-	[110, 133–135]	-	[136, 137]	-	No
	AAJT-Truncal	-	-	-	[46]	-	-	No
	TPOD	-	-	[107, 108]	[138]	[111, 136, 137]	-	No
	SAM-Sling	-	-	[109]	[138]	[111, 139, 140]	-	No
	Pelvic Binder	-	-	[110]	[138, 141]	[111]	-	No
	Resuscitative thoracotomy	[66, 70–72, 74]	-	[61, 62]	-	-	[95]	Yes
	REBOA	[116]	-	[54, 60–62, 98]	-	-	[95, 113, 115]	Yes

**Table 2:** List of identified prehospital treatment options

Class	Adjunct	Name	Manufacturier	Pro’s	Con’s
Junctional tourniquets	CRoC™	Combat Ready Clamp™	Combat medical systems. Fayetteville, NC	Easy. 4 h max	Dislodgement, heavy, limb ischemia, longer application time than other JT’s
	JETT™	Juntional Emergency Tourniquet Tool™	North American Rescue Products. Greer, SC	Easy. 4 h max. Stabilizes pelvis	No reports of clinical use
	SAM-JT™	SAM Junctional Tourniquet™	SAM Medical Products. Wilsonville, OR	Easy. 4 h max. Stabilizes pelvis	Little experience
	AAJT™	Abdominal Aortic and Junctional Tourniqet™	Compression Works. Hoover, AL	Axillary, inguinal and truncal application. External compression of the abdominal aorta	Easily broken. Uncomfortable in truncal application. Truncal max 1 h. In penetrating trauma CI for truncal placement
Wound clamp	iTC™	iTClamp™	Innovative Trauma Care. San Antonio, TX	Very easy, any location	Only superficial seal, hematoma
Haemostatic agent	XStat-30™	XStat-30™	RevMedx. Wilsonville, OR	Easy, tailored for deep penetrating wounds	Only junctional, removal of sponges
REBOA	9-14 Fr	Coda™ Balloon Catheter	Cook Medical, Indianapolis, IN	Proximal control, raises central aortic pressure	Invasive, requires physician, risk at (spinal) ischemia, challenging, time consuming
	7 Fr	ER-REBOA™	Pryor Medical. Arvada, CO		
Intra-abdominal gas insufflation	-	-	-	Minor invasive. Less risk at pressure necrosis than foam.	Abdominal compartment syndrome, risk of air embolisms, experimental
Intra-abdominal self-expanding foam	ResQFoam™	ResQFoam™	Arsenal Medical. Waterland, MA	Tamponade of abdominal compartment, less invasive then RT/REBOA, no physician required	Pressure necrosis, abdominal compartment syndrome, needs surgical removal, experimental
Pelvic stabilizer	T-POD™	Trauma Pelvic Orthotic Device™	Pyng Medical. Richmond, Canada	Easy applicable, wide experience, enables REBOA/AAJT	Insufficient if origin of haemorrhage is arterial
	SAM-sling™	SAM-sling™	SAM Medical Products. Wilsonville, OR		
	PelvicBinder™	PelvicBinder™	PelvicBinder Inc. Dallas, TX		
Pelvic Sheet	-	-	-	Low cost, widely available	Inadequate application, dislodgement
Resuscitative Thoracotomy	-	-	-	Very proximal control, raises central aortic pressure.	Invasive, risk at infection, requires physician

### Junctional haemorrhage – groin

Like in axillary haemorrhage, extremity tourniquets are not applicable in groin/inguinal or proximal lower extremity bleeding. Military guidelines advocate application of haemostatic gauze and direct manual or digital pressure in expectation of a junctional tourniquet [33].

#### Junctional haemorrhage – groin – junctional tourniquets

**Fig. 3:**
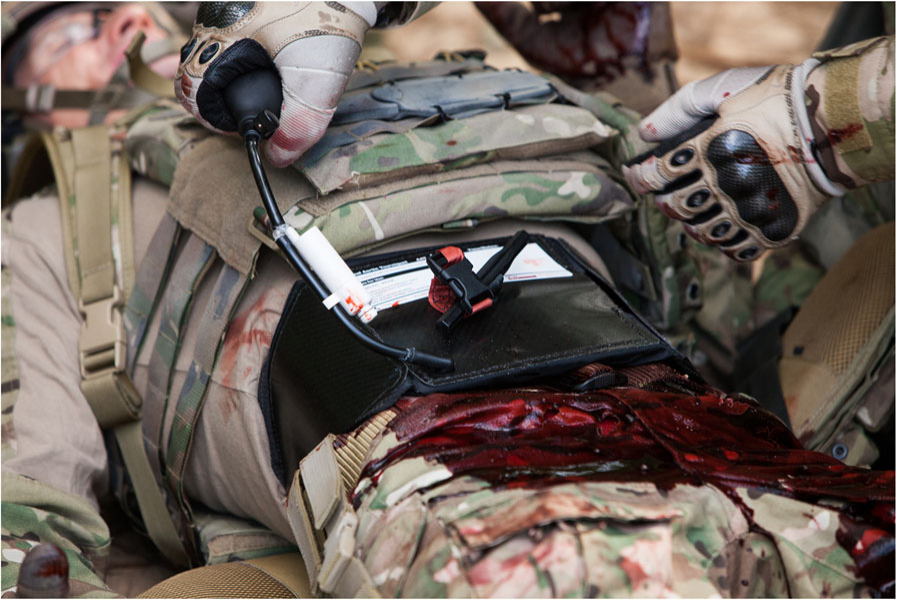
AAJT™. Image provided by manufacturer

The JETT™ and SAM-JT™ can be used in bi- or unilateral inguinal or lower extremity haemorrhage. The CRoC™ is a unilateral device. Previously, the AAJT™ had only FDA clearance for umbilical application, which occludes the abdominal aorta and can be used for bilateral inguinal or pelvic haemorrhage [43, 44]. See Fig. 3. The clearance has been expanded to junctional use: placement over the groin provides unilateral occlusion of arterial flow at the common femoral artery [40].

The CRoC™ has been reported to be effective in clinical, cadaver, manikin and animal studies in groin bleeds. See Table 1. Tovmassian et al. reported a case using the CRoC™ successfully in a proximal lower extremity traumatic amputation on the battlefield. Haemorrhage was controlled rapidly after assembling and applying of the CRoC™ which took 90 s [45]. Potential dislodgement during combat situations or transport has been mentioned as a concern of CRoC™ use [46].

**Fig. 4:**
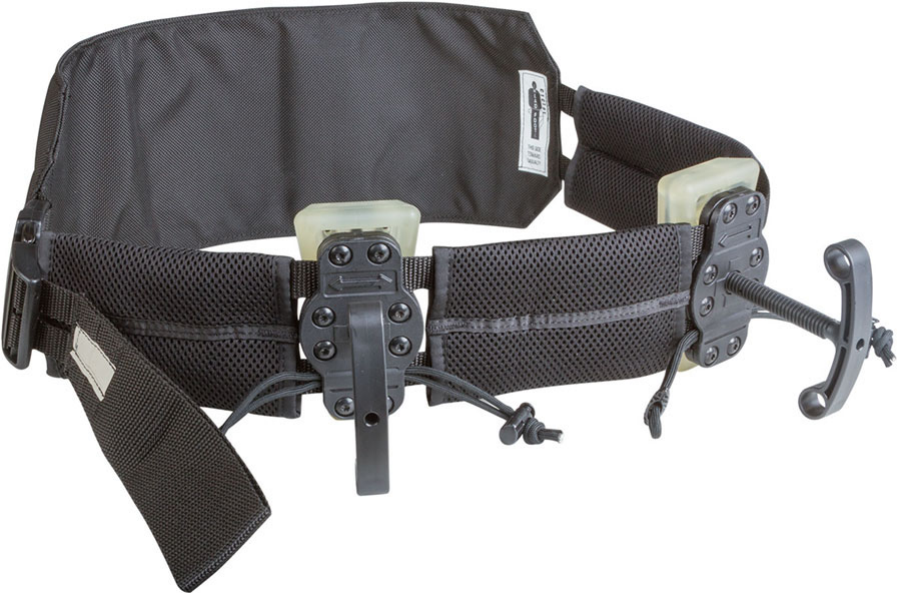
JETT™. Image provided by manufacturer

The JETT™ is designed as a belt including two pressure pads with threaded T-handles. See Fig. 4. It stabilises the pelvic ring and provides the option of bilateral haemorrhage control by compressing the common femoral artery, thereby occluding arterial blood flow to the lower extremities. Application time to occlude bilateral flow in a cadaveric study was about 10 s with JETT™. In contrast, bilateral CRoC™ application (two devices) took 68 s [47].

**Fig. 5:**
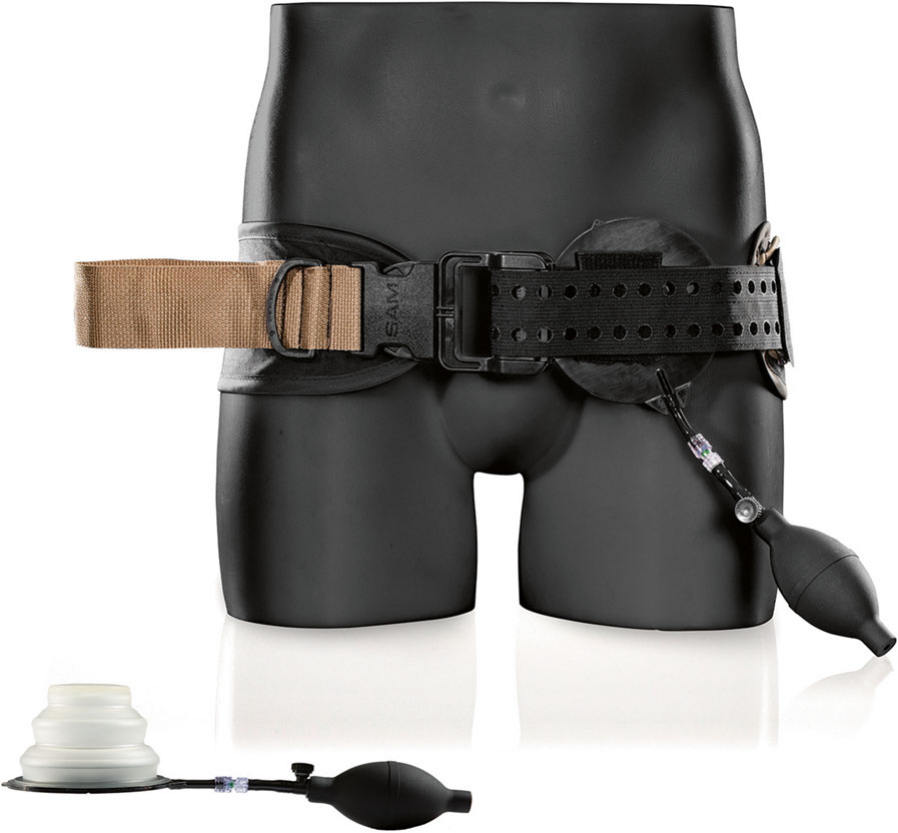
SAM-JT™. Image provided by manufacturer

The design of the SAM–JT™ is similar to the JETT™: designed like a belt, but instead of threated T-handles, the SAM-JT™ is equipped with two pneumatically inflatable bladders to compress the common femoral artery uni- or bilateral. See Fig. 5. Klotz et al. reported the use of the SAM-JT™ in inguinal bleeding of an Afghan soldier. In combination with haemostatic gauze, haemorrhage was adequately stopped [48].

The Abdominal Aortic and Junctional Tourniquet™, formerly known as Abdominal Aortic Tourniquet™, can be used in several ways. We previously mentioned the use of the AAJT™ in axillary bleeding. The AAJT™ can also be placed over the groin and has a truncal application compressing the distal aorta in the infra-renal zone at the umbilical region. In this way, bilaterally arterial blood flow to the lower extremities and to the pelvic region is blocked. Successful usage of junctional as well as truncal application has been reported in the military setting [49, 50]. Truncal application in animals was found to have minimal side effects when used for 30 min. Pregnancy, abdominal aneurysm and penetrating abdominal trauma are considered to be contraindications for truncal use of the AAJT™ [44].

Kragh et al. compared the four junctional tourniquets in a simulated out-of-hospital situation by army medics. Unfortunately, at the time of the study, the AAJT™ was only FDA-cleared for truncal application. U.S. armed forces medics preferred the CRoC™ and SAM-JT™ over the JETT™ and (truncal) AAJT™ if they could bring one device on a mission, after testing the various designs at each other [43]. Kotwal et al. reported feedback from battlefield use. The AAJT™ was reported “to be easily broken” and the CRoC™ as “bulky, heavy and takes too much time to apply” but evacuation units carry it pre-assembled in their (air) vehicles [33].

#### Junctional haemorrhage – groin – wound clamp

The iTClamp™ has been proven to be feasible in swine and perfused cadaver models with lethal femoral artery bleeding [51, 52]. Two case reports in which the iTClamp™ stopped inguinal or proximal lower extremity bleeding were published in respectively non-combatant military and civilian cases [41, 53] See Table 1.

#### Junctional haemorrhage – groin – aortic balloon occlusion

Resuscitative endovascular balloon occlusion of the aorta (REBOA) is an intervention that shows promising results in patients with non-compressible haemorrhage of the torso [54]. Endovascular balloon occlusion of the aorta was first described by Hughes to control an intra-abdominal bleeding in the operating room during the Korean war [55]. REBOA was abandoned after the high complication rate of 35 % reported by Gupta et al. in 1989. Several cases of paraplegia, thrombosis and the catheter exiting the aorta were found [56]. A recent publication of a 60 min lasting thoracic deployment of REBOA in 16 swine resulted in 4 cases of spinal ischaemia, of which 2 were lethal [57]. REBOA requires femoral arterial access for the introduction of a catheter which, after inflation, occludes the aorta. This leads to a decreased blood flow at the site of injury (distally from the balloon), and secondly to an increased central aortic pressure (CAP) [58]. Stannard et al. published an article describing a step-by-step procedure of REBOA use [59]. Zones for balloon deployment are the thoracic Zone I (left subclavian artery to coeliac artery) and zone III (infra renal aorta), for intra-abdominal or pelvic/inguinal haemorrhage respectively [59]. Several series of emergency department (ED) use of REBOA have been reported, but predominantly in cases of pelvic haemorrhage [54, 60–62]. The first prehospital REBOA intervention was performed by the London Helicopter Emergency Medical Service (HEMS) and will be discussed in the paragraph on pelvic haemorrhage. To facilitate prehospital REBOA, smaller (Fr7) balloon catheters and contrast-enhanced ultrasonic control for adequate positioning have been developed and tested in preclinical animal studies [63, 64]. Usage of REBOA to control junctional haemorrhage from the groin and lower extremities has not been described but may deem feasible.

### Intra-thoracic haemorrhage

#### Intra-thoracic haemorrhage - resuscitative thoracotomy

Resuscitative thoracotomy (RT) is a radical intervention which can be performed by any physician that can handle a scalpel [65, 66]. At the appropriate timing, RT enables relief of cardiac tamponade, aortic cross-clamping, pulmonary hilar clamping or twisting and internal cardiac compression [65–69]. Recent studies suggest that pre-hospital RT in case of penetrating trauma, more specifically stab wounds, could benefit that subcategory of patients [70]. Chance of survival for patients with gunshot wounds [65, 71] or blunt trauma are poor [65, 70, 72]. The London Helicopter Emergency Medical Service reported prehospital (or field) thoracotomy in 71 patients resulting in thirteen patients (18 %) to survive after additional treatment in a trauma centre. All survivors (*n* = 13) had a cardiac tamponade and a ventricular (*n* = 12) or aortic (*n* = 1) wound [70]. Clamshell incision (CI) may be a superior approach over left anterolateral thoracotomy since it offers better exposure and does not take more time for inexperienced physicians performed in a cadaver model for surgical residents [73]. In the prehospital case series reported by Coats et al. and Davies et al. RT was exclusively done via CI [70, 74]. Morrison et al. stated that pre-hospital thoracotomy in the military setting is futile with an estimated 0.7 % survival for patients with multiple gunshot wounds [75]. A case series of 34 prehospital thoracotomies in blunt trauma by Japanese HEMS showed no survivors [72]. A recent systematic review on emergency department resuscitative thoracotomy (EDT) in Europe showed a far more favourable survival rate of 12.9 % (18 out of 139) in blunt and 41.6 % (37 out of 89) in penetrating injuries [76].

Guidelines by the European Resuscitation Council (ERC) and Eastern Association for the surgery of Trauma (EAST) strongly recommend RT after penetrating trauma with witnessed signs of life (SoL) or ECG activity after short-term, and conditionally recommend RT in penetrating trauma without these features. In blunt trauma without witnessed SoL, RT is considered futile. Patients with blunt trauma with vital SoL and witnessed cardiac arrest RT can be performed. Nevertheless, in this ‘favourable’ group of blunt trauma patients the outcome is poor with an estimated survival rate of 1.6 % [77, 78].

### Intra-abdominal haemorrhage

Traumatic intra-abdominal haemorrhage is challenging due to the variety of organs and vessels possibly ruptured or penetrated. Rapid surgical intervention is currently the only option to stop the bleeding in case of catastrophic abdominal haemorrhage [79]. Resuscitative thoracotomy (RT) with cross-clamping of the thoracic aorta is a radical intervention in case of imminent circulatory arrest due to abdominal bleeding [65, 80, 81]. Successful case series of this intervention in the prehospital situation have been described by the London HEMS [70, 71] and by Madrid’s SAMUR-Protección Civil [66]. A laparotomy in the field is not feasible since the source of the bleeding is unknown, surgical expertise is absent and no operating theatre can be realised. In order to reduce the rate of prehospital exsanguination, research into further interventions to stop intra-abdominal bleeding are warranted [54, 82, 83].

#### Intra-abdominal haemorrhage - gas insufflation

Animal studies showed reduced blood loss by raising abdominal pressure by gas insufflation in case of hepatic [84, 85], splenic [86, 87] and inferior vena cava bleeding [88]. See Table 1. Kasotakis et al. described the possibility to use this technique en-route by using a portable carbon dioxide insufflator [87]. No clinical studies were identified for this review.

#### Intra-abdominal haemorrhage - self-expanding foam

Another invention to counter intra-abdominal haemorrhage is to inject biocompatible self-expanding foam intraperitoneally. This foam expands, engulfs the organs and becomes solid, thereby tamponading the bleeding. So far several animal studies have been executed in hepatoportal [82, 83, 89, 90], splenic [91] and iliac artery [92] models of intra-abdominal haemorrhage. See Table 1. Localized enteric pressure necrosis has been reported as frequent complication, requiring seromuscular sutures or resection [91]. Rago et al. stated that in a survey of 3442 injured soldiers in the Operation Iraqi Freedom suffering intra-abdominal bleeding, 34 % of patients required bowel repair and 19 % even resection anyway, so the localized necrosis might be considered an acceptable complication in patients facing imminent exsanguination [91, 93]. In July 2015, Mesar et al., published a study on the optimal human dosage by injecting the foam in recently deceased humans [94]. At the time of this review, no clinical use of ResQFoam™ (Arsenal Medical, Watertown MA) has been published.

#### Intra-abdominal haemorrhage - REBOA

The previously described REBOA can also be applied to thoracic zone (Zone I), thereby occluding abdominal aortic flow in case of intra-abdominal haemorrhage. REBOA is considered to be far less invasive compared to a thoracotomy with descendent aortic cross clamping [95]. It has been assessed in various animal models [57, 58, 63, 96]. as well as in the clinical setting [54, 61, 62, 97]. See Table 1. Ogura et al. published a case series of patients (*n* = 8) with haemoperitoneum after blunt abdominal trauma with a successful in-hospital REBOA in 86 % (*n* = 7) [97]. Moore et al. reported a lower mortality rate for in-hospital REBOA (*n* = 24) vs resuscitative thoracotomy (*n* = 72) in non-compressible truncal haemorrhage: 62.5 % vs 90.3 %. Injury Severity Score did not differ significantly. However, in the RT-group a higher percentage received cardiopulmonary resuscitation (CPR) upon arrival [62]. Analysing patients from the Japanese trauma data bank who received an in-hospital REBOA, Norii et al. found a higher mortality in patients receiving a REBOA compared to those who did not, even after correction for injury pattern and other variables. This might be explained by REBOA being used as a ‘last ditch’ effort. Interestingly, REBOA is part of the Japanese emergency physicians arsenal since trauma surgeons are not always attending 24/7 [98].

#### Intra-abdominal haemorrhage - aortic abdominal and junctional tourniquet (AAJT)™

The AAJT™ has not been reported for abdominal bleeding, however when no other options are available, its use might be feasible since it has the ability to restrict the intra-abdominal compartment by external pressure, theoretically providing an earlier tamponade of the abdominal cavity. Nevertheless, abdominal application is contra-indicated in the case of penetrating injury [46].

#### Pelvic haemorrhage

Pelvic fracture, or the so-called ‘the killing fracture’ [99], is potential lethal, especially in severe pelvic disruption with following haemorrhagic shock [100]. Mortality of traumatic pelvic injury is estimated at 28 % by Papakostidis et al. in a recent systematic review [101]. Haemodynamic instability is present in 10 % of pelvic fractures [102], and often the origin of the bleeding is uncertain: it may be caused by (a combination of) bleeding from fracture surfaces, the venous plexus or arteries [103].

A study of 5340 German trauma patients with a pelvic fracture showed a mortality of 4 % (238 patients). In these deceased patients, massive haemorrhage was the cause of death in 34 %, of which 62 % was specified to the pelvic region. Holstein et al. did not include prehospital mortality, so the mortality rate due to exsanguination is likely to be even higher [104].

#### Pelvic haemorrhage - circumferential binders

Several devices providing circumferential pressure are on the market [103], if not equipped with such a device a conventional sheet should be applied [105]. Placing a circumferential binder (CB) or sheet will reduce the volume of the intra-pelvic cavity, compress the fracture surfaces and prevent dislodgement of newly formed clots by preventing second displacement [106].

Tan et al. reported a reduced symphyseal diastasis of 60 % in unstable pelvic fractures after emergency department application of a Trauma Pelvic Orthotic Device™ (T-POD™, Pyng Medical, Richmond Canada) and a rise in mean arterial pressure form 65.3 to 81.2 [107]. Croce et al. stated that the implementation of the T-POD™ significantly reduced transfusion requirement (9.9 vs 21.5 units) and length of stay (16.5 vs 24..4 days) [108]. The SAM-sling™ (SAM Medical Products, Oregon, USA) is an alternative, and was tested clinically by Krieg et al. in 16 patients with a pelvic ring injury upon arrival at the ED resulting in an 9.9 % reduction of the pelvic distension [109]. The PelvicBinder™ (PelvicBinder Inc., Dallas, USA) has been reported to be superior over sheet wrapping for emergency stabilisation of the pelvis [110]. Knops et al. found all of the three commercial temporary pelvic stabilizers providing sufficient reduction in of the pelvic fracture in a cadaveric study, with the T-POD requiring the lowest force [111].

If the patient haemodynamically further deteriorates, despite application of a CB, an arterial origin is suspected [112]. The CB should be left in place, and an AAJT™ can be placed at the umbilical region to externally compresses the abdominal aorta to the spine, prohibiting distal arterial flow to the pelvis and lower extremities. Taylor et al. reported successful aortic occlusion in 15 of 16 voluntary soldiers by Doppler sound examination of the common femoral artery [46].

#### Pelvic haemorrhage- REBOA

REBOA in uncontrolled pelvic haemorrhage is of great interest and both preclinical [113–115] and clinical publications on this topic are vast [54, 60–62, 116]. See Table 1. As the iliac artery is on the route of the REBOA placement, unilateral femoral pulsation is required. An in-hospital series of 13 cases of uncontrollable haemorrhage due to pelvic injury the use of REBOA led to initial haemorrhage control in 12 patients. Deflation of the balloon in the angiographic suite resulted in recurrence of haemodynamic instability in half of the patients. Ultimately 6 of the initial patients survived to discharge. The Injury Severity Scores (ISS) differed among survivors and non-survivors (ISS 38 versus 58, *p* = 0.011) but the Revised Trauma Score did not: 4.362 versus 4.779, *p* = 0.761 [60]. Brenner et al. reported ED deployment of REBOA within 6 min (range 4–6) in a series of 6 patients (5 cases of pelvic fracture and 1 patient with a renal injury). Two patients did not survive due to concomitant brain injury and non-survivable injuries [54]. The London HEMS in 2014 reported the first pre-hospital performed REBOA in a patient with a pelvic fracture with a successful control of the bleeding bridging transport to definitive care [116].

## Discussion

This study provides an overview of established, novel and future prehospital treatment options in care for trauma patients with exsanguinating truncal or junctional haemorrhage. Some options have been incorporated in protocols worldwide, i.e., temporary pelvic stabilizers [105], as others, such as REBOA [117], have regained new interest after they were previously abandoned. Throughout history, war necessitated innovative medical improvements [28, 118]. The development of junctional tourniquets, for instance, is the result of changes in injury patterns in the current conflicts. An increase of blast injuries from improvised explosive devices (IED’s) resulted in a combination of pelvic fracture, traumatic lower limb amputations and torso injuries [119] requiring more proximal haemorrhage control than can be provided by extremity tourniquets [33].

Unfortunately, nowadays civilians in western societies are increasingly victim of penetrating trauma due to shootings, stabbings and bombings. Especially in mass casualty scenarios from recent (terrorist) attacks, victims show injury patterns that resemble those in the military setting [120, 121]. Evaluation of the 2013 Boston Marathon bombing revealed that knowledge from the military (i.e., the use of an extremity tourniquet) was not yet translated to civilian prehospital trauma care and that further progress is desirable [122]. Recently the *Hartford Consensus* promotes application of tourniquets and usage of haemostatic gauze dressings by non-(para) medical responders and lay persons to stop bleeding early [29–31].

As many of the novelties described were developed from a military medicine point of view experience, it is worthwhile to discuss whether the innovations might be useful for the civilian prehospital arena [28, 123]. Military prehospital care may differ from civilian prehospital care in several ways: patient population, trauma mechanism, on-going gunfire, remoteness and skill level of care providers. Combat casualties sustain a much higher ratio of penetrating and blast injuries in contrast to the mainly blunt injuries in civilian patients. However, soldiers are likely to be fitter than the average civilian population, and more able to compensate in case of haemorrhagic shock. In general, EMS teams encounter less hostile working circumstances than military medics [123]. Furthermore, extraction time and/or transport to the nearest facility for damage control (forward surgical team facility) in general takes longer than in the civilian situation (trauma centre). However, in the setting of civilian cases in remote and/or rural area’s rescue and transport times are usually prolonged mimicking the military situation [124]. So, in these cases when confronted with massive haemorrhage, achieving early haemorrhage control on scene or during transport might result in a better chance at survival when the nearest adequate definitive care facility is obvious too far away to justify ‘scoop and run’ [2, 6, 125]. Moreover, it may not be feasible to maintain direct pressure (as primary treatment of junctional bleeding) during evacuation. In a study on the feasibility of manual pressure, 44 clinicians had to provide bimanual compression at a maintainable effort: mean pressure was 39 kg (range 17–60). This was found to be insufficient to occlude flow in the iliac and abdominal aortic artery which required pressure of 54 kg and 63 kg respectively [126].

A 2015 survey on the translation of innovations in military medicine to civilian practice revealed military experience was of importance to implementation of massive transfusion protocol (63 %), tourniquets (60 %) and haemostatic gauzes (41 %) in US civilian trauma systems. Overall, inventions proven effective in military medicine were considered effective ‘most of the time’ (52 %) or ‘always’ (10 %) in the civilian setting! However, civilian research confirming military data on the inventions was considered insufficient [28].

High quality clinical studies in the population of haemorrhagic trauma patients in extremis are clearly difficult to conduct. Preclinical studies in a standardized model, such as the swine model described by Kheirabadi et al. [127], should form the basis for the development of new adjuncts. For non-invasive options (i.e., junctional tourniquets), testing in a simulation model in volunteers would be the next step [43]. Feedback from the field in small pilot studies by military or prehospital civilian care-providers will provide for further direction of development [33, 43]. As pointed out by Haider et al. collaboration of civilian and military physicians is essential provide evidence in how life-threatening haemorrhage can be dealt with in the (chaotic) prehospital arena [28].

Several interventions discussed in this review require high skill level of the prehospital care provider. For the performance of prehospital REBOA or prehospital resuscitative thoracotomy, a physician-staffed prehospital team is required. Application of junctional tourniquets, iTClamps™, X-stat™, pelvic circumferential binders and topical haemostatics can be applied by trained EMS paramedics. Possible future options as intra-abdominal insufflation or self-expanding foam may be appropriate to be used by paramedics.

Successful implementation of interventions for truncal and/or junctional bleeding in civilian prehospital care systems requires (trauma) surgical leadership or similar involvement. Careful study of the efficacy of the concerned interventions is mandated but literature is yet limited to small case series. Analysis of the prehospital trauma patient population (in the EMS catchment area) is mandated regarding trauma mechanism, incidence of massive haemorrhage, rescue and transport time to assess the meaningfulness of implementation of a certain intervention. For instance, in case of an urban system, short (i.e., less than 15 min) transport times to definitive care facilities may deem interventions as REBOA unwanted but this may not be the case when the victim has to be retrieved from a remote area. Nevertheless even in an urban system definitive care can be delayed by longer prehospital times caused by entrapment, confinement or even heavy traffic. Also, e.g., in a rare civilian case of junctional bleeding, direct pressure (when enough personnel available) during transport might be the best option instead of training and equipping all ambulance personnel and ambulances with junctional tourniquets. There are no data on patients that might have had a hypothetical benefit from prehospital junctional and truncal haemorrhage control in civilian context situation so the incidence, from the in-hospital point of view point, seem low. Nevertheless, from a conceptual point of view, early haemorrhage control is of vital importance. Advanced prehospital haemorrhage control (i.e., junctional tourniquets, REBOA, resuscitative thoracotomy, etc.) may be considered as an additional expert task for physician-staffed EMS when timely dispatched to the patient at the scene or the ambulance; diminishing blood loss and transfusion requirements.

The trade-off of a more aggressive prehospital haemorrhage control system with the use of the retrieved adjuncts is the concomitant consequence of side-effects and/or complications. See Table 2. Since the devices are designed to stop bleeding, ischaemia is an important complication. X-stat and the junctional tourniquets have to be removed within 4 h after application [33, 35]. The truncal placement of the AAJT has an ultimatum of one hour [44, 46]. Due to the large pressure the AAJT effectuates over the abdomen, some effect on ventilation may occur [33]. REBOA has a serious risk of spinal or mesenteric ischaemia and therefore application has to be as short (20–40 min) as possible [61, 96, 117].

Life-threatening haemorrhage from extremities has been impressively tackled by prehospital application of extremity tourniquets, and focus will shift to stop junctional and truncal haemorrhage. This review aimed to provide an comprehensive overview of established, state-of-the-art and future strategies to arrest catastrophic haemorrhage in order to bridge time to definitive care in patients who formerly died of exsanguination prior to reaching a trauma centre. Unfortunately the level of evidence is extremely limited, but awareness of potentially life-saving novelties is a first step to encounter the increasing threat of exsanguination from combat-like injuries in the civilian arena. Indiscriminate implementation of the discussed interventions is not advisable since there is scarce data on potential harmful side-effects (e.g., due to ischaemia). Moreover, one should be aware of the risk of “indication gliding” which is seen in many other medical fields - a device developed for life-threatening situation start being used in less injured patients, and the risk of side-effects become more important.

## Conclusion

Control of bleeding from junctional areas and non-compressible torso bleeding remains the greatest challenge in prehospital trauma care. Junctional tourniquets, resuscitative thoracotomy, REBOA, pelvic circumferential binders and the iTClamp™ have been developed. Injury pattern and transport time to definitive care differs significantly between combat care and civilian trauma care. In case of bomb attacks, stabbings, mass shootings, civilian prehospital situations resemble military settings. The use of military inventions such as tourniquets and haemostatic gauze dressings have been successfully translated to the civilian situation. The focus of research will now probably shift to stopping junctional and truncal bleeding. Several promising treatment options have been developed, but overall the level of evidence of the included studies is extremely low.

## Abbreviations

AAJT: Abdominal aortic and junctional tourniquet
AAT: Abdominal aortic tourniquet
CAP: Central aortic pressure
CB: Circumferential binder
CRoC: Combat ready clamp
EAST: Eastern Association for the surgery of Trauma
ED: Emergency department
EDT: Emergency department thoracotomy
EMS: Emergency medical service
ERC: European resuscitation council
HEMS: Helicopter emergency medical service
IED: Improvised explosive device
ISS: Injury severity score
ITC: iTClamp
JETT: Junctional emergency tourniquet tool
REBOA: Resuscitative endovascular balloon occlusion of the aorta
RT: Resuscitative thoracotomy
SAM-JT: SAM junctional tourniquet
SOL: Signs of life
